# Long non‐coding RNA LUCAT1 promotes tumourigenesis by inhibiting ANXA2 phosphorylation in hepatocellular carcinoma

**DOI:** 10.1111/jcmm.14088

**Published:** 2018-12-26

**Authors:** Yun Lou, Yue Yu, Xiaolia Xu, Shu Zhou, Haiyuan Shen, Tianlong Fan, Di Wu, Jie Yin, Guoqiang Li

**Affiliations:** ^1^ Department of Liver Surgery The First Affiliated Hospital of Nanjing Medical University Nanjing Jiangsu province China; ^2^ Key Laboratory of Living Donor Transplantation of Ministry of Public Health Nanjing Jiangsu province China; ^3^ Medical School of Southeast University Nanjing Jiangsu province P.R. China; ^4^ Department of Respiratory Medicine Jinling Hospital Nanjing Jiangsu province China; ^5^ Department of Hepatobiliary Surgery of Drum Tower Clinical Medical College Nanjing Medical University Nanjing China; ^6^ Department of Hepatobiliary Surgery The Affiliated Drum Tower Hospital of Nanjing University Medical School Nanjing China

**Keywords:** Annexin A2, hepatocellular carcinoma, long non‐coding RNA, phosphorylation, tumour progression and metastasis

## Abstract

Long non‐coding RNAs (lncRNAs) play essential roles in diverse biological processes; however, current understanding of the mechanism underlying the regulation of tumour proliferation and metastasis is limited. Lung cancer‐associated transcript 1 (LUCAT1) has been reported in a variety of human cancers, while its role in hepatocellular carcinoma (HCC) remains unclear. This study aimed to determine the biological role and underlying mechanism of LUCAT1 on progression and metastasis in HCC cells and clinical specimens. Our results demonstrated that LUCAT1 was up‐regulated in HCC tissues and cells. Loss‐ and gain‐of‐function studies revealed that LUCAT1 promotes the proliferation and metastasis of HCC cells in vitro and in vivo. Furthermore, RNA pulldown and Western blot assays indicated that LUCAT1 inhibited the phosphorylation of Annexin A2 (ANXA2) to reduce the degradation of ANXA2‐S100A10 heterotetramer (AIIt), which in turn accelerated the secretion of plasminogen into plasmin, thereby resulting in the activation of metalloprotease proteins. In conclusion, we propose that LUCAT1 serves as a novel diagnostic and therapeutic target for HCC.

## 1 INTRODUCTION

Hepatocellular carcinoma (HCC) is one of the most common cancers in adults, accounting for one‐fifth of the incidence of malignant tumours in China.^1^, ^2^ Its main pathogenic factors include chronic hepatitis B infection, chronic alcohol abuse and non‐alcoholic fatty liver disease.^3^ Despite advances in the understanding of the molecular mechanisms underlying progression and therapeutic treatments for HCC, the median survival rate remains at approximately 50% (17%‐69%) after 5 years.^4^ This poor prognosis is mainly led by the frequent intrahepatic or extrahepatic metastasis of HCC. Cancer classification using biomarkers may define the risk of recurrence, which provides for the use of appropriate treatments for a better prognosis. But to date, few measurable biomarkers for predicting HCC metastasis or therapeutic targets for HCC have been identified.

Long non‐coding RNAs (LncRNAs) are a class of poorly conserved non‐coding RNA transcripts that are longer than 200 nucleotides.^5^ It is now clear that various LncRNAs can function as signals, decoys, guides or scaffolds for other regulatory proteins.^6^, ^7^, ^8^, ^9^ For example, the LncRNA HULC promotes the phosphorylation of YB‐1 through the extracellular signal‐regulated kinase pathway, which then regulates the interaction of YB‐1 with certain oncogenic mRNAs, thereby accelerating their translation to promote tumourigenesis.^10^ Additionally, the LncRNA TSLNC8 exerts its tumour suppressive activity through inactivation of the IL‐6/STAT3 signaling pathway by physically interacting with TKT and STAT3, which then inhibits STAT3 phosphorylation and its transcriptional activity in HCC.^11^ These studies indicate that despite various ways through which LncRNAs affect the development of HCC, a part of LncRNAs can interact with proteins to regulate post‐translational modifications such as phosphorylation or ubiquitination, ultimately influencing their activities and functions.

Annexin A2 (ANXA2) is a pleiotropic calcium‐ and anionic phospholipid‐binding protein that exists as a monomer and as heterotetrameric complex with the plasminogen receptor protein, S100A10.^12^ The complex is also named as AIIt. Annexin A2 has been proposed as an important part in many processes, including plasmin generation and matrix invasion.^13^, ^14^, ^15^ It is proven that ANXA2 stabilizes S100A10, which in turn directly participates in binding to tPA and plasminogen and enhancing plasminogen activation. Plasminogen is then converted into its active proteolytic form plasmin. Plasmin mediates the hydrolysis and remoulding of the extracellular matrix (ECM), and activates metalloprotease (MMPs) such as MMP‐9 12.

Lung cancer‐associated transcript 1 was first reported to be involved in smoking‐related lung cancer and was previously called smoke and cancer‐associated LncRNA1 (SCAL1).^6^ It is also associated with non‐small lung cancer, oesophageal squamous cell carcinoma and colorectal cancer and glioma.^7^, ^8^, ^9^ It is proved that LUCAT1 regulates cell proliferation via epigenetically repressing p21 and p57 expression in non‐small lung cancer.^9^ Nevertheless, the expression pattern, underlying molecular mechanisms and biological roles of LUCAT1 in HCC tumourigenesis remain unclearly defined. In the present study, we found that LUCAT1 was up‐regulated in tumour tissues compared to adjacent non‐cancerous tissues and correlated with tumour size, metastasis and Edmondson grade of HCC. Our results indicated that LUCAT1 promotes tumour progression and metastasis in vivo and in vitro. Furthermore, it is demonstrated in our study that LUCAT1 inhibits the phosphorylation of ANXA2 at Ser‐25 site to reduce the degradation of AIIt, which in turn accelerated the secretion of plasminogen into plasmin, thereby resulting in the activation of MMP proteins. Our results provide insights into the importance of LUCAT1 in the tumourigenesis and progression of HCC and as a predictor for survival.

## 2 METHODS

### 2.1 Patient samples and cell lines

A total of 90 paired tissues of HCC tumours and adjacent normal samples and related clinical information were obtained from patients undergoing liver resection at the Liver Transplantation Center in The First Affiliate Hospital of Nanjing Medical University between October 2012 and March 2013. This research was approved by our Institutional Ethics Committee. All patients in our study offered their informed consent to take part in our study prior to surgery. Fresh tissues were collected and immediately frozen in liquid nitrogen. The diagnosis of all patients was histopathologically confirmed. The human hepatoma cell lines, HepG2, SMMC‐7721, SNU‐423, Hep3B, Huh7, MHCC‐97H and the human normal L02 cell line were obtained from KeyGen (Nanjing KeyGen Biotech Co., Ltd., Jiangsu, China). The cells were cultured in DMEM medium (Gibco, Carlsbad, CA, USA) supplemented with 10% foetal bovine serum (FBS) and maintained in an atmosphere of 5% CO_2_ at 37°C.

### 2.2 RNA isolation and quantitative reverse transcription‐PCR

Total RNA was isolated from tissues using TRIzol and purified with the RNeasy MinElute Clean up kit (Qiagen, Hilden, Germany) according to the manufacturer’s instruction. cDNAs were synthesized from the total RNA using the random priming method. Transcript levels were measured in duplicate by quantitative reverse transcription‐PCR (qRT‐PCR) (ABI 7900; Life Technologies). Expression levels were calculated relative to that of GAPDH.

### 2.3 Transfection of cell lines

Full‐length LUCAT1 was subcloned into the lentivirus vector GV367 to overexpress LUCAT1.1 (Gene, Shanghai, China). To knock down the expression of LUCAT1, a short hairpin RNA (shRNA) sequence aiming for LUCAT1 was subcloned into the lentivirus vector pLL3.7 (Gene), and the negative control hairpin shRNA with no sequence homology to any human gene was subcloned into the control groups. For overexpressing ANXA2, sequence of full‐length ANXA2 was subcloned into the lentiviral vector GV367 (Gene). All vectors were labelled with luciferase. Transfection was conducted according to the manufacturer's instructions, and qRT‐PCR was performed to determine transfection efficiency. The cells were then subjected to RNA extraction or functional assays.

### 2.4 Cell proliferation, migration and invasion assay

Cell proliferation was assessed by the clone formation assay using a Cell Counting Kit‐8 (Dojindo Laboratories, Kumamoto, Japan) and EDU (5‐ethynyl‐2‐madeoxyuridine) immunofluorescence staining assay (Millipore, Billerica, MA, USA) according to the manufacturers’ instructions. Cell migration and invasion were determined using Transwell chambers (8 μm pore size, Millipore, Darmstadt, Germany). In the invasion assay, over the membrane, a thin layer of ECM was dried. The ECM layer occluded the membrane pores and blocked the non‐invasive cells from migrating. To the top chamber, 5 × 10^3^ cells in serum‐free medium were added. In the lower chamber, DMEM with 10% FBS was added. After 48 hours, cells that had invaded through the membrane were fixed with methanol, stained with crystal violet and counted. In the migration assay, similar protocol was performed that was used for the invasion assay described above, except that the ECM layer was not added to the chamber, and 2 × 10^3^ cells were added per chamber.

### 2.5 Wound healing assay

Cells were seeded at a density of 4 × 10^4^ cells/cm^2^. On day 3, a straight scratch was made with a 200 mL pipette tip and images of the wound were acquired under the microscope with the original magnification of 100×. After 0, 24 and 48 hour, cells were photographed under the microscope and the remaining scratch area calculated.

### 2.6 Flow cytometry analysis of cell cycle and apoptosis

To detect changes in the cell cycle upon LUCAT1 alteration, the sh‐LUCAT1‐97H, sh‐NC‐97H, sh‐LUCAT1‐Hep3B, sh‐NC‐Hep3B, Lv‐LUCAT1‐Huh7, and Lv‐NC‐Huh7, Lv‐LUCAT1‐HepG2 and Lv‐NC‐HepG2 cells were subjected to serum starvation to induce cell cycle synchronization. The cells were harvested at the logarithmic growth period and fixed in 70% ethanol overnight at −20°C. The next day, the cells were stained with propidium iodide (PI) and analysed by flow cytometry. For apoptosis analysis, the cells were cultured in a complete medium with 0.5 mmol/L peroxide overnight, double stained with Annexin V and PI using the FITC Annexin V Apoptosis Detection Kit (Roche, Basel, Switzerland) according to the manufacturer’s instructions, and analysed by flow cytometry.

### 2.7 In vivo experiments

Male BALB/c nude mice (6‐8 weeks old) were purchased from the Animal Center of Nanjing University (Nanjing, Jiangsu, China), raised and maintained in permitted animal facilities at Nanjing Medical University, and the experiments were conducted in accordance with the guidelines approved by Animal Studies Committee. In the subcutaneous transplantation model, four mice were implanted with sh‐LUCAT1‐MHCC97H cells (5 × 10^6^ cells suspended in 200μL phosphate buffered saline [PBS]) in the left groin and sh‐NC‐MHCC97H (5 × 10^6^ cells suspended in 200μL PBS) in the right groin. The volume of the tumours were calculated every five days after transplantation using the equation V = 0.5 × D×d^2^ (V, volume; D, longitudinal diameter; and d, latitudinal diameter). Forty days after injection, the mice were killed and the corresponding tumours were collected. In the lung metastasis model, mice (eight in each group) were injected with cells (5 × 10^6^ cells suspended in 200μL PBS) via the tail vein. One group was injected with Lv‐LUCAT1‐HepG2 cells, and the other with Lv‐NC‐HepG2 cells, which were all labelled with luciferase. The mice were killed 6 weeks after injection, and hematoxylin and eosin (H&E) staining of the lung tissues was performed to verify metastasis of the HCC cells and to calculate the number of tumours.

### 2.8 RNA binding protein immunoprecipitation

According to the manufacturer's instructions, we used the special antibody for RNA binding protein immunoprecipitation (RIP) assays that specifically targeted ANXA2. The co‐precipitated RNAs were detected by RT‐PCR. To ensure that the detected signals were from the RNAs specifically binding to ANXA2, negative controls and input controls were performed as well.

### 2.9 RNA pulldown assays

For RNA pulldown assays, the biotin‐labelled LUCAT1 transcript and the LUCAT1 intron sequence and its antisense were obtained using in vitro transcription with T7 RNA polymerase and Biotin RNA Labeling Mix (Roche), followed by 4 hours of incubation at 37°C with oncosphere cell lysates. Then, streptavidin‐conjugated agarose beads were used for centrifugal enrichment. The precipitated components were separated using SDS‐PAGE, followed by silver staining. Differential bands were isolated for mass spectrometry (MS) (LTQ Orbitrap XL).

### 2.10 RNA fluorescence in situ hybridization

RNA fluorescence in situ hybridization (RNA‐FISH) assays were performed as described below. Digoxygenin (DIG) labelled LUCAT1 probes were used for RNA FISH. MHCC97H cells were fixed in 4% formaldehyde and permeabilized with 0.5% Triton X‐100 for 5 minutes, washed with PBS three times and once in 2X SSC buffer. Hybridization was carried out using DNA probe sets at 37°C for 12 hours. Images were obtained with a FV1000 confocal laser microscope (Olympus).

### 2.11 Northern blot analysis

LUCAT1 levels were measured by northern blotting using an Ambion Northern Max‐Gly Kit (Austin, TX, USA). The total RNA was electrophoresed on a 1% agarose gel and was siphoned to a positively charged nylon membrane (NC). The RNA was then fixed to the NC membrane using UV crosslinking. The cross‐linked membrane was pre‐hybridized with Ultrahyb‐oligo hybridization buffer and hybridized with the LUCAT1‐specific and GAPDH‐specific oligonucleotide probes labelled with DIG‐ddUTP in roller bottles.

### 2.12 Western blot assay

Tissue samples and cultured cells were incubated with RIPA reagent plus phenylmethanesulfonylfluoride (Beyotime, Nantong, China) to isolate the proteins. Approximately 30 mg of total proteins were loaded into each lane and then transferred onto PVDF membranes. The membranes were incubated at 4°C overnight with human‐specific antibody of ANXA2, p‐Ser25‐ANXA2 and Tubulin (Abcam, London, UK) and S100A10, E‐cadherin, N‐cadherin, VIM, Snail CyclinD1 and P27 (CST, Boston, MA, USA). Hybridization was visualized using the chemiluminescent detection system (Pierce ECL substrate western blot detection system; Thermo Scientific, Waltham, MA, USA).

### 2.13 Statistical analysis

All experimental assays were performed in triplicate. The data were expressed as the mean ± SEM. Differences between two independent groups were tested with the student’s *t* test using spss 19.0 (spss, Palo Alto, CA, USA) and presented with GraphPad Prism 5.0 (GraphPad Software, La Jolla, CA, USA). Kaplan‐Meier survival curves were plotted and log‐rank test was done. The significance of various variables for survival was analysed by Cox proportional hazards model in a multivariate analysis. *P* value <0.05 was considered statistically significant.

## 3 RESULTS

### 3.1 LUCAT1 is overexpressed in HCC tissues

**Figure 1:**
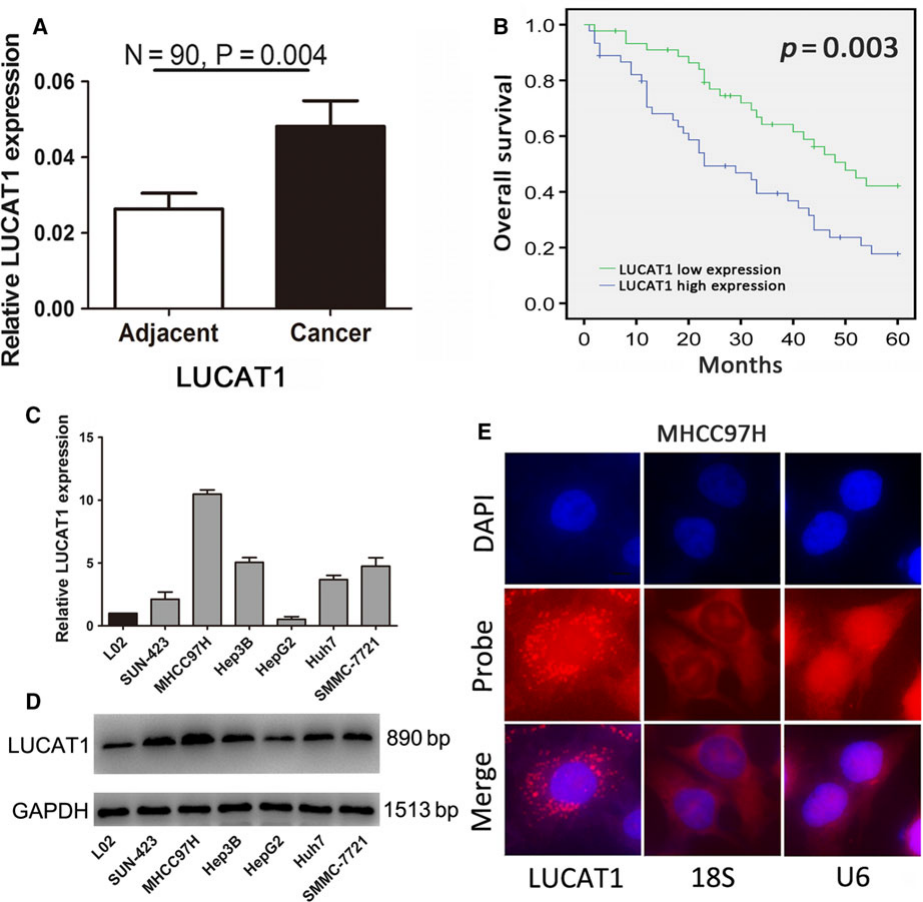
Lung cancer associated transcript 1 (LUCAT1) is aberrantly up‐regulated in HCC tissues. (A) An increase in the expression of LUCAT1 occurs in hepatocellular carcinoma (HCC) tumour tissues compared to matched adjacent non‐tumour tissues (n = 90). Lung cancer associated transcript 1 expression was tested by quantitative real‐time PCR and measured using the $2^{-\Delta \Delta C_{T}}$ method, analysed with paired student's *t* test, and presented as means ± SEM. (B) Based on the median value of the LUCAT1 expression in HCC tissues, patients were divided into two groups (LUCAT1‐high expression group and LUCAT1‐low expression group), the Kaplan‐Meier survival analysis was used to calculate the overall survival. (C,D) The differential expression level of LUCAT1 in HCC cells, detected by quantitative reverse transcription‐PCR and northern blot assays. (E) RNA fluorescence in situ hybridization was conducted to detect the sub‐location of LUCAT1 (red) in MHCC97H cells, which revealed that it was mainly located in cytoplasm. A scale is presented at the lower right of the first panel. Magnification: 400×

To confirm the elevated expression of LUCAT1 in HCC tissues, we first examined its expression levels in 90 pairs of liver cancer and adjacent non‐cancerous tissues by qRT‐PCR. An increase in LUCAT1 expression was found in the HCC samples (*P = *0.004; Figure 1A). To further explore the clinical significance of the aberrant expression of LUCAT1 in HCC, according to the bimodal expression pattern of LUCAT1 in HCC patients (Figure S1A), we separated all the HCC patients into two groups: LUCAT1‐high expression group and LUCAT1‐low expression group based on the median value of LUCAT1. Statistical analysis showed that LUCAT1 expression is highly associated with tumour size, metastasis and tumour node metastasis (TNM) stage but not sex, age, HBsAg, cirrhosis and tumour number (Table 1). Additionally, survival analysis showed that HCC patients with high LUCAT1 expression levels had poorer prognosis than those with low LUCAT1 expression and a shorter overall survival (OS) time (Figure 1B). Furthermore, univariate analysis showed that metastasis (*P < *0.001), poor/undifferentiated grade (*P < *0.001) and high expression of LUCAT1 (*P = *0.006) were significant risk factors for poorer prognosis. Furthermore, metastasis (Yes/No, heart rate (HR) 2.275, 95% CI 1.091‐4.747, *P = *0.028), Edmondson grade (I‐II/III‐IV, HR 0.367, 95% CI 0.172‐0.785, *P = *0.010) and LUCAT1 expression (high/low, HR 3.692, 95% CI 1.471‐9.295, *P = *0.007) were found to be independent prognostic factors by multivariate analysis (Table 2).

**Table 1:** Correlation between LUCAT1 expression and clinicopathological characteristics of HCC patients (n = 90)

Characteristics	Total	LUCAT1 low expression (<median^a^)	LUCAT1 high expression (≥median^a^)	p Chi‐squared test *P‐*value
n	90	45	45	
Sex				0.267
Male	59	27	32	
Female	31	18	13	
Age (y)				0.288
<60	40	17	22	
≥60	50	28	23	
HBsAg				0.396
Negative	15	9	6	
Positive	75	36	39	
Cirrhosis				0.598
Absent	18	10	8	
Present	72	35	37	
Tumour size (cm)				0.003*
≤5	61	37	24	
>5	29	8	21	
Tumour number				0.238
Single	65	35	30	
Multiple	25	10	15	
Metastasis				0.001*
Yes	19	3	16	
No	71	42	27	
Edmondson grade				0.003*
I‐II	63	38	25	
III‐IV	27	7	20	

HCC, hepatocellular carcinoma; LUCAT, lung cancer associated transcript 1.

a The median expression level of LUCAT1 was used as the cut‐off.

* *P*‐value < 0.05.

**Table 2:** Univariate and multivariate survival analyses evaluating LUCAT1 expressing in HCC

Characteristics	Univariate analysis		Multivariate analysis	
	Log rank χ^2^ test	*P*	HR（95% CI）	*P*
Sex	0.005	0.945		NI
Male				
Female				
Age (y)	0.584	0.447		NI
<60				
≥60				
HBsAg	0.078	0.781		NI
Negative				
Positive				
Cirrhosis	0.007	0.931		NI
Absent				
Present				
Tumour size (cm)	0.853	0.358		NI
≤5				
>5				
Tumour number	0.263	0.610		NI
Single				
Multiple				
Metastasis	21.605	0.000*		0.028*
Yes			Reference	
No			2.275 (1.091‐4.747)	
Edmondson grade	30.091	0.000*		0.010*
I‐II			Reference	
III‐IV			0.367 (0.172‐0.785)	
LUCAT1 expression	8.084	0.006*		0.007*
High			Reference	
Low			3.692 (1.417‐9.295)	

HCC, hepatocellular carcinoma; LUCAT, lung cancer associated transcript 1.

* *P*‐value < 0.05.

### 3.2 LUCAT1 promotes proliferation and metastasis of HCC cells in vitro

**Figure 2:**
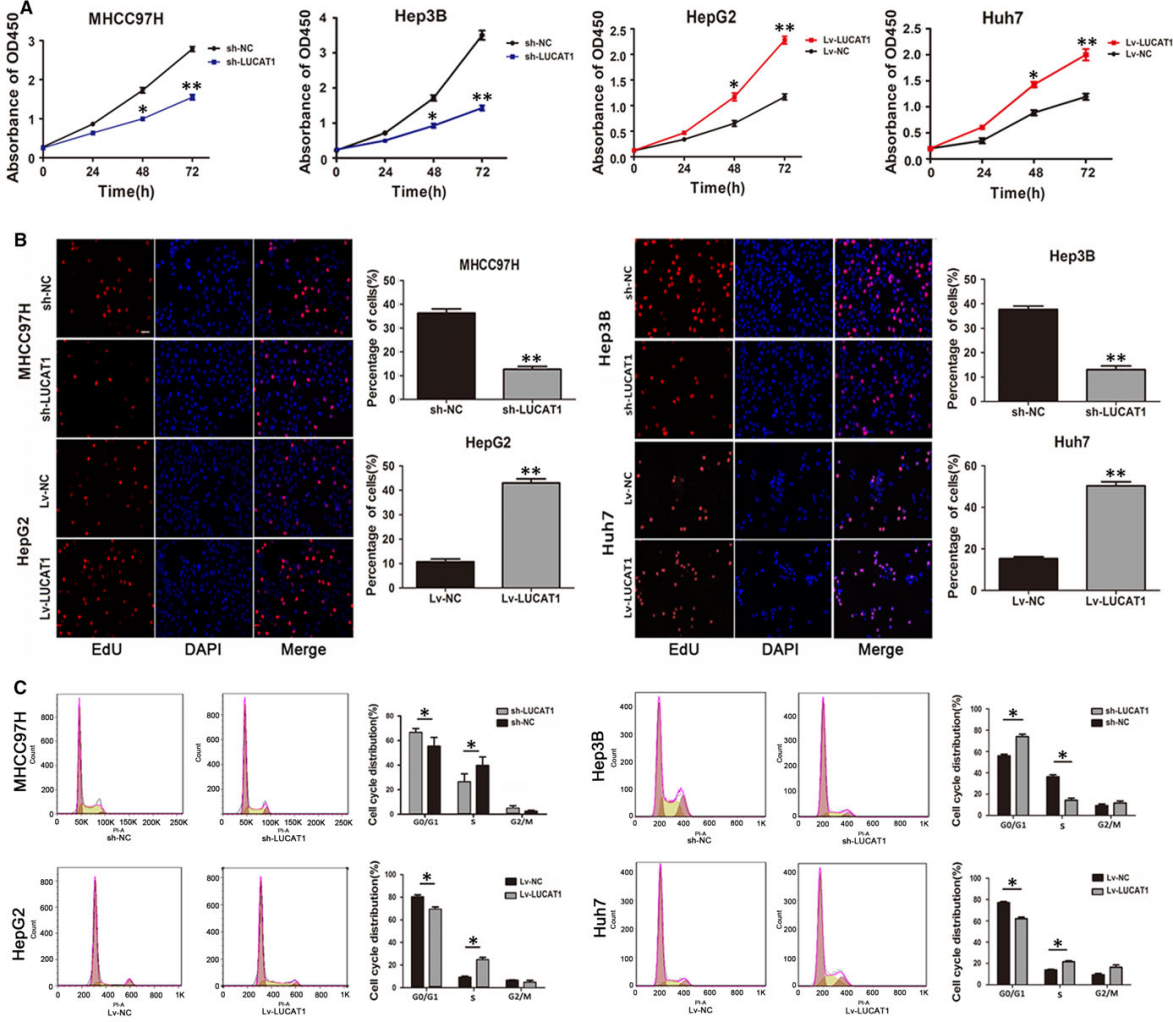
Lung cancer associated transcript 1 (LUCAT1) enhances the proliferation of hepatocellular carcinoma (HCC) cells in vitro. (A) Proliferation rates were assessed using the CCK8 assay, and overexpression of LUCAT1 promoted HepG2 and Huh7 cell proliferation, whereas knockdown of LUCAT1 inhibited MHCC97H and Hep3B cell proliferation. (B) EdU immunofluorescence staining confirmed the function of LUCAT1 on HCC cell proliferation. Magnification: 100×. (C) The cell cycle distribution of HCC cells treated with LUCAT1 was assessed by flow cytometry. The distribution of cell cycle is shown in the graphs. All experiments were performed in triplicate and expressed as the mean ± SEM (*P < 0.05, ** *P* < 0.01)

To investigate the biological functions of LUCAT1 in vitro*, *we detected the expression levels of LUCAT1 in the HCC cell lines, human normal liver cell LO2 as a control. The results of qRT‐PCR and northern blot assays showed that the expression of LUCAT1 is significantly higher in the HCC cell lines compared to the LO2 cells, especially MHCC97H cells (Figure 1C,D). Based on the RNA expression of LUCAT1 in the HCC cell lines, we detected the subcellular location of LUCAT1 by probe hybridization in MHCC97H cells. The LUCAT1 probe revealed that LUCAT1 mainly located in cytoplasm (Figure 1E). Next, we selected the MHCC97H and Hep3B cell line to conduct LUCAT1 knock down, and HepG2 and Huh7 cell line to conduct LUCAT1 overexpression, all detected by qRT‐PCR and northern blot assays(Figure S1B,C). To explore the influence of LUCAT1 on cell proliferation, we conducted CCK8 and EdU assays on the HCC cell lines. The CCK8 assays showed that the Lv‐LUCAT1‐HepG2 cells, as well as Lv‐LUCAT1‐Huh7 cells, have increased proliferation ability compared to the control groups, whereas that of the sh‐LUCAT1‐MHCC97H cells and sh‐LUCAT1‐Hep3B cells were suppressed, indicating that LUCAT1 promotes HCC cell growth (Figure 2A). Furthermore, EdU assays at the 48 hour time‐point also confirmed the more vigorous proliferation (Figure 2B). These findings suggest that LUCAT1 accelerates HCC cells proliferation, which may via controlling cell cycle progression and cell apoptosis ability. Thus, flow cytometry experiments were conducted to assess cell cycle progression, which showed a reduction in the G0/G1 population and an elevation in the S‐phase population in Lv‐LUCAT1‐HepG2 and Lv‐LUCAT1‐Huh7 cells, whereas LUCAT1 knockdown assay showed the opposite effects in the MHCC97H and Hep3B cells (Figure 2C). However, the results of the cell apoptosis assays showed no evidence of an association between LUCAT1 and cell apoptosis (Figure S1D).

**Figure 3:**
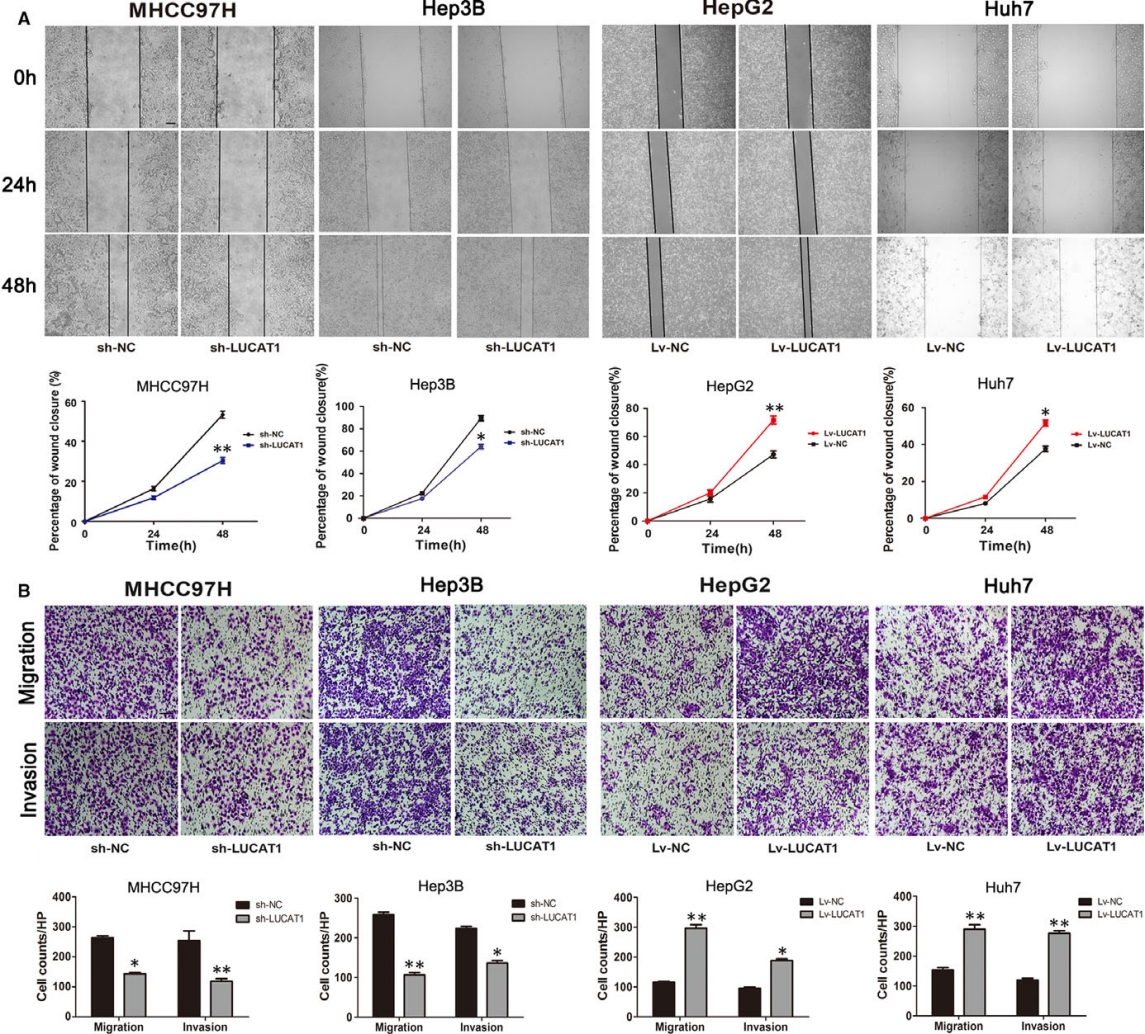
Lung cancer associated transcript 1 (LUCAT1) enhances hepatocellular carcinoma (HCC) cell migration and invasion in vitro. (A) Wound healing assays were conducted to assess HCC cell migration. Representative wound healing images at 0, 24 and 48 h are shown. Quantitative analysis revealed that LUCAT1 significantly enhances HCC cell migration. Magnification: 40×. (B) Transwell assays of overexpressed and silenced LUCAT1 and their negative control HCC cells were performed, and migration and invasion were measured. The bar graph shows the number of cells that migrated or invaded the membrane. Magnification: 100×. All experiments were performed in triplicate and expressed as the mean ± SEM (*P < 0.05, ** *P* < 0.01)

Correlation analysis between LUCAT1 and clinicopathological characteristics implied that LUCAT1 might participate in the regulation of HCC metastasis. To investigate whether LUCAT1 is involved in the modulation of invasion and migration abilities of HCC cells, wound healing and Transwell assays were performed. Wound closure was significantly suppressed in the sh‐LUCAT1‐MHCC97H cells compared to the sh‐NC‐MHCC97H cells, and in the sh‐LUCAT1‐Hep3B cells compared to the sh‐NC‐Hep3B cells, whereas that in the Lv‐LUCAT1‐HepG2 cells closed faster than the Lv‐NC‐HepG2 cells, with same appearance in Huh7 cells (Figure 3A). The Transwell assays showed a significant increase in the migration and invasion abilities of the Lv‐LUCAT1‐HepG2 cells compared to the Lv‐NC‐HepG2 cells, and of the Lv‐LUCAT1‐Huh7 cells compared to the Lv‐NC‐Huh7 cells. Knockdown of LUCAT1 in MHCC97H and Hep3B cell lines sharply decreases its migration and invasion abilities (Figure 3B).

### 3.3 LUCAT1 enhances tumour growth and metastasis in vivo

**Figure 4:**
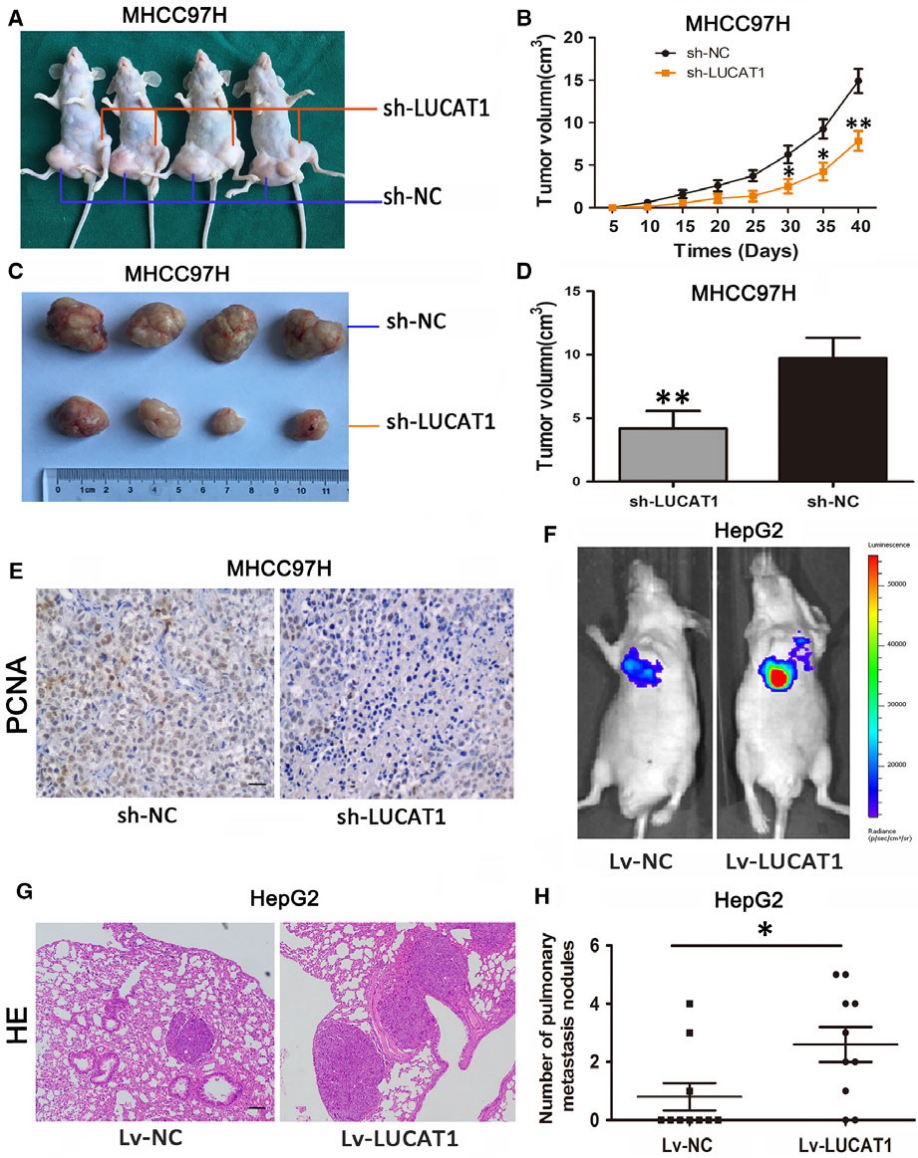
Lung cancer associated transcript 1 (LUCAT1) enhances tumour growth and metastasis in vivo. (A‐F) BALB/c nude mice (6 wk of age) were subcutaneously transplanted with MHCC97H cells, sh‐LUCAT1‐MHCC97H cells into the left groin and sh‐NC‐MHCC97H cells into the right groin (four mice in each group). The volume of the tumours was calculated every 5 d after transplantation, and the mice were killed 40 d after implantation. Lung cancer associated transcript 1 strengthened MHCC97H cell growth in the tumours of the nude mice, which was confirmed by the staining of formalin‐fixed paraffin‐embedded of the subcutaneous tumour tissues with proliferating cell nuclear antigen (PCNA) Magnification: 200×. (F) In the tail vein xenograft model, mice (10 in each group) were injected with HepG2 cells (5 × 106 cells suspended in 200 μL phosphate buffered saline) through the tail vein, and lung metastasis was investigated respectively using the IVIS Lumina II system. (G) All the results of lung colonization were validated by the histological examination (H&E) Magnification: 100×. (H) Compared to the control group (three mice presented lung colonization), eight mice showed lung colonization with a higher number and larger tumours in the LUCAT1 overexpressing group. (*P < 0.05, ** *P* < 0.01)

To further determine the functional effects of LUCAT1 on tumourigenesis in vivo, a xenotransplantation model was built, in which nude mice were subcutaneously injected with MHCC97H cells stably knocked down for LUCAT1 or a control vector. The left groin was injected with sh‐LUCAT1‐MHCC97H cells, and the right groin was injected with the control cells. The xenografts produced from LUCAT1 knocked down cells showed significantly decreased tumour growth compared to the controls, and the final tumour volume was smaller than the controls (Figures 3A‐D). The staining of formalin‐fixed paraffin‐embedded (FFPE) subcutaneous tumour tissues with proliferating cell nuclear antigen (PCNA) confirmed the lower proliferation status of LUCAT1 knockdown cells (Figure 4E). To further investigate the lung metastasis of HCC cells, we constructed a tail vein xenograft model by injecting Lv‐LUCAT1‐HepG2 cells and Lv‐NC‐HepG2 cells separately into the two groups of nude mice and each group contained 10 mice. The results revealed lung colonization in the LUCAT1 overexpressed group with eight mice presenting lung colonization at the end of the experiment, whereas only three mice established lung colonization in the control group, with fewer and smaller tumours (Figures 4F,H). All the lung colonization of HCC cells was validated by histological examination (Figure 4G). Taken together, these results reveal that LUCAT1 promotes growth and metastasis of HCC cells in vivo.

### 3.4 LUCAT1 interacts with ANXA2

**Figure 5:**
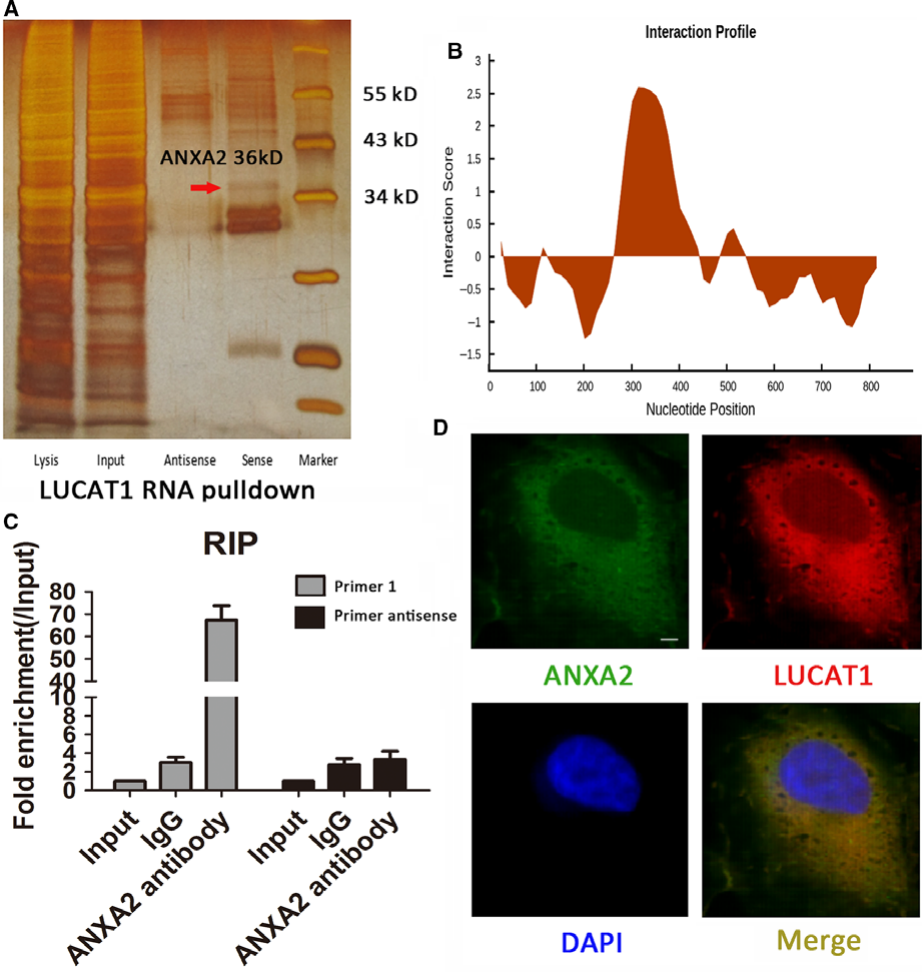
Lung cancer associated transcript 1 (LUCAT1) interacts with Annexin A2 (ANXA2). (A) RNA pulldown assays were performed using biotin‐labelled sense or antisense LUCAT1 in MHCC97H cells. Sliver staining and mass spectrometry were conducted to identify the interacting proteins. The red arrow indicates the ANXA2 band. (B) Schematic map of the potential binding site of LUCAT1 on the ANXA2 protein. (C) RNA binding protein immunoprecipitation (RIP) assays were performed using an anti‐ANXA2 antibody and confirmed with agarose gel electrophoresis using a different probe. Fold increases were calculated by comparing with the input in the lower panel. (D) Antibody‐labelled ANXA2 (green) and probelabelled LUCAT1 (red) was detected in the MHCC97H cells. DAPI (blue) staining indicates nuclei. A scale is presented at the lower right of the first panel. Magnification: 400×. Data are expressed as the means ± SEM

Many studies reveal that LncRNAs can regulate the function of proteins through RNA‐protein interactions.^16^ We used RNA pulldown assays followed by silver staining and MS to identify LUCAT1‐binding proteins. Annexin A2 was found to specifically bind to LUCAT1 (Figure 5A). The binding site of LUCAT1 and ANXA2 was predicted using the RNA sequence of LUCAT1 and the amino acid sequence of ANXA2 as input in Cat‐Rapid database. We found an enrichment region encompassing 315 to 366 nt of the RNA sequence of LUCAT1 (Figure 5B). RIP assay using the ANXA2 antibody was also conducted. We designed two independent primers of LUCAT1 for PCR assays after hybridization (primer 1 targeting the 315 to 366 nt region, whereas primer 2 was used as control). The binding status of LUCAT1 and ANXA2 was confirmed (Figure 5C). Moreover, RNA‐FISH and immunofluorescence assays indicated that LUCAT1 colocalized with ANXA2 in the cytoplasm of HCC cells (Figure 5D). Because previous studies have proven that ANXA2 plays an essential role in tumourigenesis and progression of HCC,^17^, ^18^, ^19^, ^20^ we propose that LUCAT1 exert its function through the LUCAT1/ANXA2 axis in HCC cell lines.

### 3.5 LUCAT1 inhibits the phosphorylation of ANXA2 and accelerates the secretion of plasminogen into plasmin

**Figure 6:**
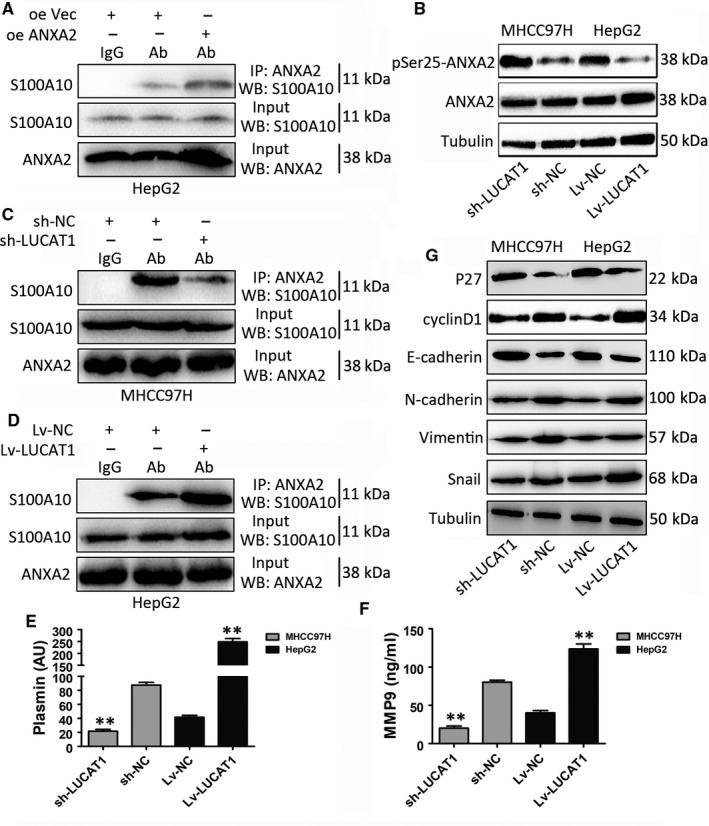
Lung cancer associated transcript 1 (LUCAT1) inhibits the phosphorylation of Annexin A2 (ANXA2) to accelerate plasminogen into plasmin. (A) Co‐immunoprecipitation (Co‐IP) assays were performed with S100A10 antibody in ANXA2 overexpressing cells. (B) Annexin A2 and pSer25ANXA2 expression levels were investigated in LUCAT1 overexpressed and deleted cells. (C,D) Co‐immunoprecipitation assays showed that ANXA2 precipitated less S100A10 in LUCAT1 knockdown cells (C) and more S100A10 in LUCAT1 overexpressing cells (D). (E,F) ELISA assays revealed the influence of LUCAT1 on the expression of plasmin and MMP‐9. (G) epithelial–mesenchymal transition‐ and cell cycle‐related proteins were investigated in LUCAT1 overexpressed and deleted cells

Previous studies have proven that ANXA2 exist as monomers or as heterotetrameric complexes with S100A10 (P11), and the ANXA2/S100A10 complex is referred to as AIIt, which was known to function in adherens junction formation in epithelial^21^ and endothelial cells based on its association with epithelial E‐cadherin and endothelial VE‐cadherin.^22^ Before any further investigation, we conducted ANXA2‐overexpressing HepG2 cell lines (Figure S2A,B). Figure 6A shows the binding of ANXA2 and S100A10 in HCC cell lines using co‐immunoprecipitation (Co‐IP) assays. As LUCAT1 could bind to ANXA2, we wonder whether the expression or phosphorylation status of ANXA2 could be affected by LUCAT1 in HCC cell lines. Figure 6B shows that there was no differential change in the total protein expression of ANXA2 upon LUCAT1 alteration, but the Ser‐25 phosphorylation status of ANXA2 was apparently affected by LUCAT1. The expression of pSer25‐ANXA2 decreased in LUCAT1‐overexpressing cells and increased in LUCAT1 knockdown cells. In the AIIt complex, ANXA2 played an obligatory role in the regulation of S100A10 by protecting S100A10 from rapid ubiquitin‐mediated degradation.^23^, ^24^, ^25^, ^26^ Furthermore, it has been proven that the phosphorylation of Ser‐25 of ANXA2 triggers the dissociation of the AIIt complex, which results in the release of S100A10 as it undergoes ubiquitin‐mediated proteasomal degradation.^12^, ^27^ Next, to investigate whether LUCAT1 influences the AIIt complex, we conducted Co‐IP assays, which showed that ANXA2 precipitated less S100A10 in LUCAT1 knockdown cells (Figure 6C) and more S100A10 in LUCAT1 overexpressing cells (Figure 6D).

The colocalization of the plasminogen activators and plasminogen driven by AIIt could increase the cleavage of plasminogen into plasmin, which further activates pro‐MMPs (matrix MMPs) into active MMPs.^12^, ^28^, ^29^, ^30^ Previous studies have revealed that plasmin and MMPs could function as carcinogenic factors by enhancing the proliferation and metastasis of various tumours, including HCC.^31^, ^32^, ^33^, ^34^ Next, to explore alterations in the expression of plasmin and MMP proteins caused by LUCAT1, ELISAs were conducted. Figures 6E,F show that the expression of plasmin and MMP‐9 increased when LUCAT1 was overexpressed and decreased when LUCAT1 was knocked down. Also, the classical epithelial–mesenchymal transition (EMT)‐related genes and cell cycle‐regulated genes were detected by Western blotting upon LUCAT1 alterations (Figure 6G), which showed that LUCAT1 promotes the expression of EMT‐related genes and cell cycle‐related genes in HCC cell lines.

Taken together, these results show that LUCAT1 inhibits the phosphorylation of ANXA2 at Ser‐25, and then suppresses the degradation of AIIt, thereby promoting the stable expression of AIIt on the cell surface, resulting in the generation of plasmin and subsequently activating the MMP proteins.

## 4 DISCUSSION AND CONCLUSION

Lung cancer associated transcript 1 was first reported to be involved in smoking‐related lung cancer, whereas its function in HCC remains unclear. Here, we detected the roles of LUCAT1 in HCC tumourigenesis and metastasis. With a cohort of 90 randomly selected HCC patients undergoing liver resection 5 years ago, we found an obvious association between the up‐regulation of LUCAT1 and tumour size (>5 cm, *P* = 0.003), as well as tumour metastasis (yes, *P* = 0.001) and TNM stage (Ⅲ‐Ⅳ, *P* = 0.003). Multivariate analysis showed that LUCAT1 expression level was an independent risk factor for the OS (HR 3.692, 95% CI 1.417‐9.295; *P* = 0.007) after surgery. These results suggest that overexpression of LUCAT1 might be a warning sign that post‐operative patients should be closely monitored and receive further appropriate adjuvant therapies. It is regrettable that the serum level of LUCAT1 was unable to be acquired. Further validation of the up‐regulation of LUCAT1 in serum of another set of HCC patients should be undertaken.

The HCC cells with overexpression and knockdown of LUCAT1 were then subjected into a series of assays. Our results showed that LUCAT1 promotes the progression and metastasis of HCC cells in vivo and in vitro. Next, to further detect the molecular mechanism of LUCAT1, the RNA pulldown assays were performed to explore the potential downstream target. Our results revealed that LUCAT1 bound to ANXA2 specifically. Annexin A2 is proved to be implicated in various cancers, including HCC.^17^, ^35^, ^36^, ^37^, ^38^ For example, Zhang et al reported that shRNA‐mediated silencing of ANXA2 suppresses the invasion, migration and tumourigenic potential of hepatoma cells and may thus be utilized as a target of future molecular therapies.^39^ As ANXA2 binds with LUCAT1, and considering the desperate need to find a better therapeutic target for HCC patients, we designed subsequent experiments to determine the mechanism of ANXA2 and LUCAT1.

**Figure 7:**
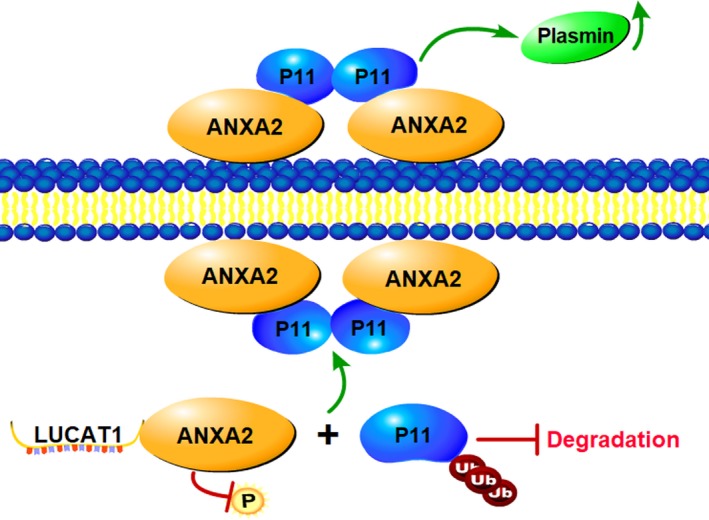
Schematic diagram illustrating that Lung cancer associated transcript 1 (LUCAT1) inhibits the phosphorylation of Annexin A2 (ANXA2) at Ser‐25, further suppressing the degradation of AIIt, and then accelerating the conversion of plasminogen into plasmin, which activates metalloprotease proteins, leading to the progression and metastasis of HCC

Interestingly, Western blot showed the expression of ANXA2 did not change in relation to alterations of LUCAT1 expression. Thus, we wonder whether LUCAT1 is involved in the post‐translational modification of ANXA2. Several studies reveal that ANXA2 has three phosphorylation sites, Ser‐11, Tyr‐23 and Ser‐25.^12^, ^40^ Out of curiosity, we detected the expression of expression of three phosphorylation sites of ANXA2 upon LUCAT1 alteration separately. The results showed decreased expression of pSer25ANXA2 when LUCAT1 was up‐regulated (Figure 6B), suggesting that LUCAT1 inhibited the phosphorylation of ANXA2 at Ser‐25, while other phosphorylation sites showed no changes likewise (data not shown). It has been proven that the phosphorylation of Ser‐25 of ANXA2 triggers the dissociation of the AIIt complex, which results in the release of S100A10 which then undergoes ubiquitin‐mediated proteasomal degradation.^12^, ^27^ Co‐immunoprecipitation assays confirmed the combination between ANXA2 and S100A10, and it is obvious that ANXA2 precipitated less S100A10 in LUCAT1 knockdown cells and more S100A10 in LUCAT1 overexpressing cells, which indicated that LUCAT1 inhibits the phosphorylation of ANXA2 at Ser‐25 site to further suppress the degradation of AIIt. He et  al reported that the colocalization of the plasminogen activators and plasminogen driven by AIIt could increase the cleavage of plasminogen into plasmin, which further activate pro‐MMPs (matrix MMPs) into active MMPs.^12^, ^28^, ^41^, ^42^ Based on these findings, we then performed ELISA assays and found that the expression of plasmin and MMP‐9 increased when LUCAT1 was overexpressed (Figure 6E,F; Figure 7), indicating that the suppression of degradation of AIIt equivalently promotes the stable expression of AIIt on the cell surface and accelerating the cleavage of plasminogen into plasmin to activate MMP proteins.

In conclusion, we revealed that LUCAT1 plays a key role in HCC tumour progression and metastasis. Our results therefore suggest that the LncRNA LUCAT1 might be a predictive marker for HCC metastasis and target for drug development.

## CONFLICT OF INTEREST

The authors declare that they have no financial conflict of interest.
