# Supplementary figures and images for: Long non‐coding RNA LUCAT1 promotes tumourigenesis by inhibiting ANXA2 phosphorylation in hepatocellular carcinoma
Source: J Cell Mol Med. 2018 Dec 26;23(3):1873–84. doi: 10.1111/jcmm.14088 (PMC6378214; doi:10.1111/jcmm.14088)

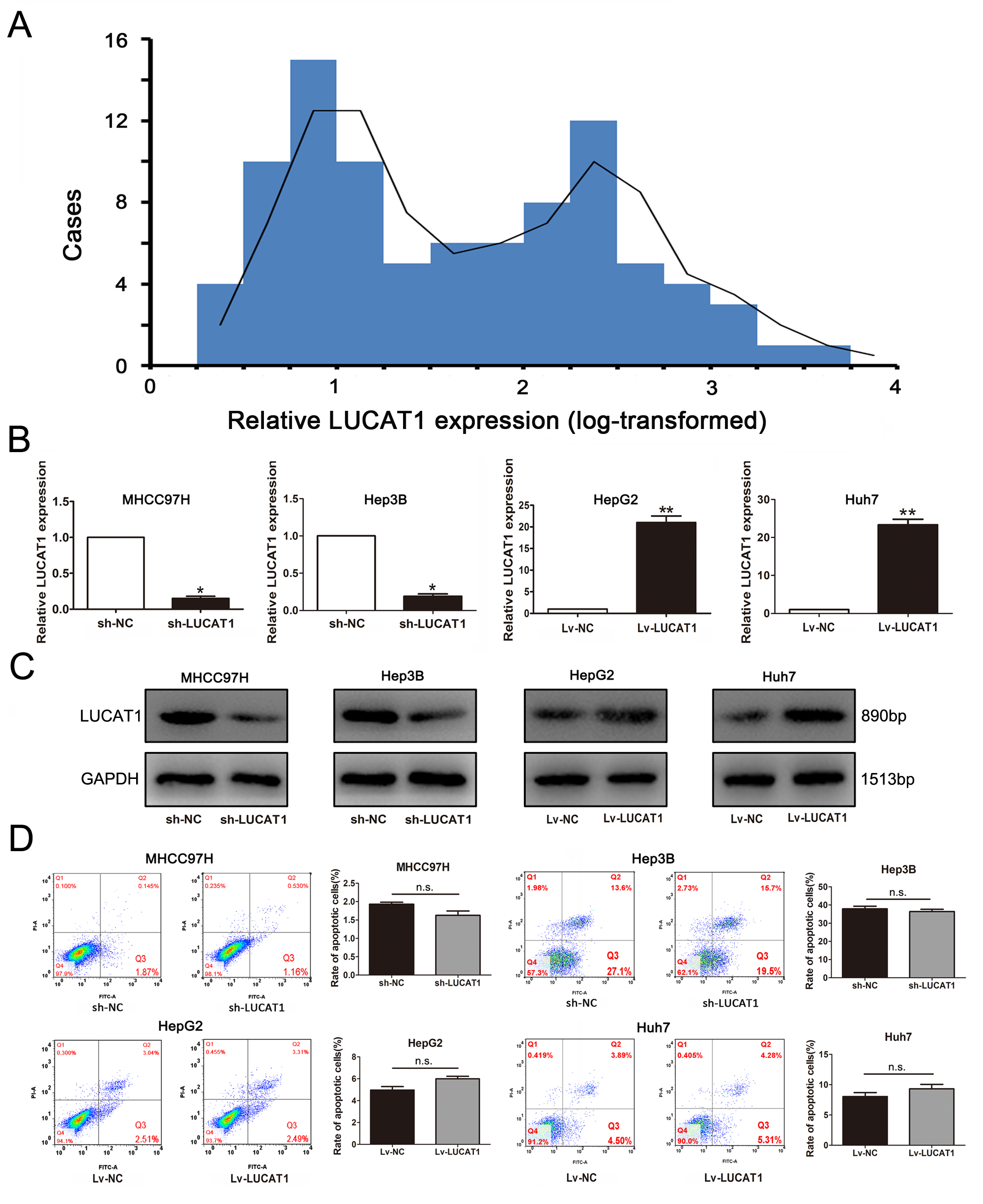

Supplement: Supplementary file 1 [file JCMM-23-1873-s001.tif]

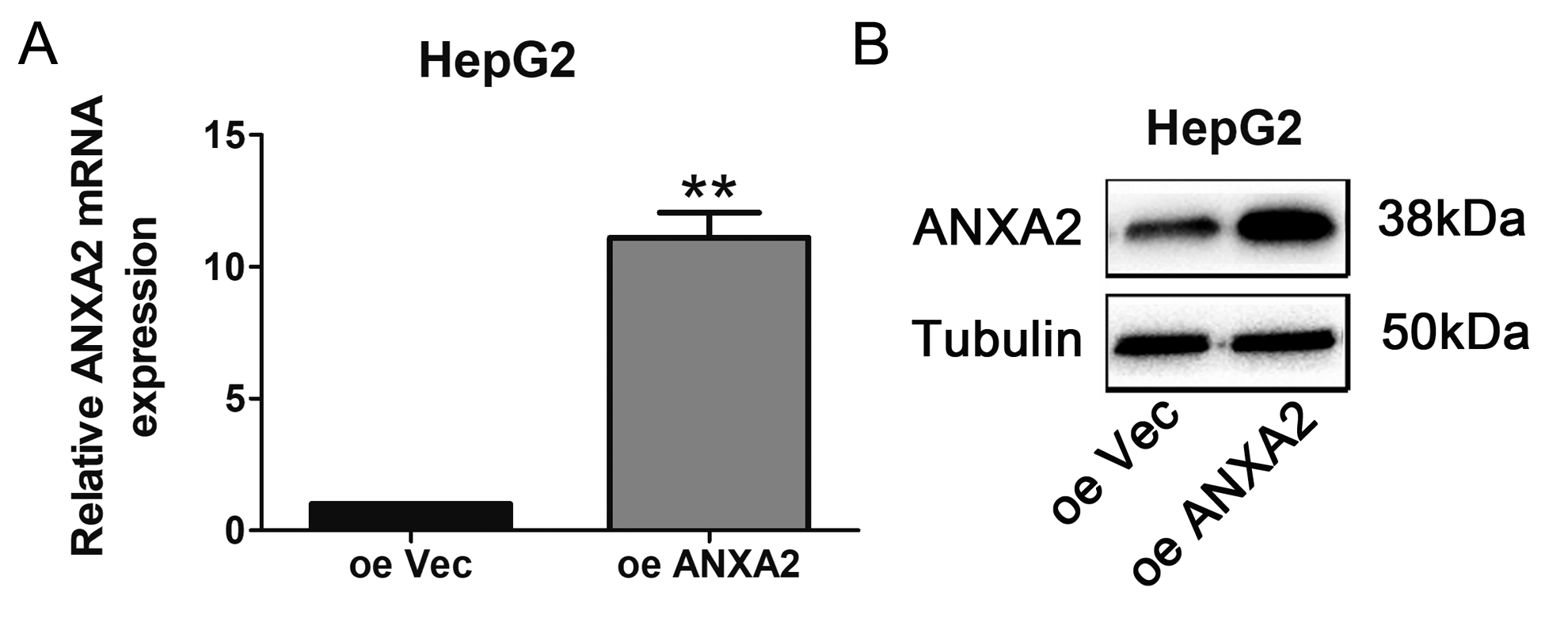

Supplement: Supplementary file 2 [file JCMM-23-1873-s002.tif]
