# Supplementary material for: Long non‐coding RNA LUCAT1 promotes tumourigenesis by inhibiting ANXA2 phosphorylation in hepatocellular carcinoma
Source: J Cell Mol Med. 2018 Dec 26;23(3):1873–84. doi: 10.1111/jcmm.14088 (PMC6378214; doi:10.1111/jcmm.14088)
Supplement: Supplementary file 3 [file JCMM-23-1873-s003.docx]

**Supplementary Figure 1:** The distribution of the expression of LUCAT1 in HCC patients, and the transfection efficiency and cell apoptosis assays in LUCAT1-overexpressing and -deleted cells.

**Supplementary Figure 2:** The transfection efficiency in ANXA2-overexpressing cells.
